# Assessment of murine lung mechanics outcome measures: alignment with those made in asthmatics

**DOI:** 10.3389/fphys.2012.00491

**Published:** 2013-02-12

**Authors:** Julia K. L. Walker, Monica Kraft, John T. Fisher

**Affiliations:** ^1^Division of Pulmonary, Allergy and Critical Care Medicine, Duke University Medical CenterDurham, NC, USA; ^2^Physiology Program, Department of Biomedical and Molecular Sciences, Queen's UniversityKingston, ON, Canada; ^3^Division of Respirology, Department of Medicine, Queen's UniversityKingston, ON, Canada

**Keywords:** airway hyperresponsiveness, murine, asthma, lung mechanics, translational research

## Abstract

Although asthma is characterized as an inflammatory disease, recent reports highlight the importance of pulmonary physiology outcome measures to the clinical assessment of asthma control and risk of asthma exacerbation. Murine models of allergic inflammatory airway disease have been widely used to gain mechanistic insight into the pathogenesis of asthma; however, several aspects of murine models could benefit from improvement. This review focuses on aligning lung mechanics measures made in mice with those made in humans, with an eye toward improving the translational utility of these measures. A brief description of techniques available to measure murine lung mechanics is provided along with a methodological consideration of their utilization. How murine lung mechanics outcome measures relate to pulmonary physiology measures conducted in humans is discussed and we recommend that, like human studies, outcome measures be standardized for murine models of asthma.

## Asthma control

Asthma is a chronic inflammatory disorder of the airways characterized by paroxysmal or recurring symptoms that are associated with variable airflow obstruction and bronchial hyperresponsiveness (Lougheed et al., 2012). Structural changes to the airways often occur and this remodeling may be associated with persistent disease and a progressive decline in lung function.

Given the heterogeneity and complex pathophysiology underlying asthma, disease progression should be carefully assessed and closely monitored to achieve successful management. Goals of asthma management per the National Asthma Education Prevention Program (NAEPP) guidelines include minimization of asthma symptoms, optimization of lung function, and prevention of exacerbations (NAEPP, 2007). Simply put, the goal of asthma treatment is to “control” the disease. The NAEPP and a more recent guideline by the American Thoracic Society (ATS) (Reddel et al., 2009) define asthma control as “the extent to which the various manifestations of asthma have been reduced or removed by treatment.” In other words, effective asthma treatment should hinder the progression of the disease from mild to moderate and moderate to severe and reduce the frequency and severity of asthma “attacks,” thereby reducing impairment and future risk.

The definition of asthma has not changed much over the last few decades; however, how asthma is assessed and how these assessments correlate to the progression of the disease has recently undergone modification. Recognizing the importance of “asthma control” to quality of life, the ATS recently assembled a task force to provide consensus recommendations to standardize the definition of, and assessment methods for, asthma control (Reddel et al., 2009). In brief, whereas the term “asthma control” was once defined by an examination of a patient's recent clinical state [including symptoms, reliever use, lung function, and airway hyperresponsiveness (AHR)], it now includes an assessment of future risk. Similar to the NAEPP guidelines, the ATS guidelines recommend that both clinical state and future risk be assessed to characterize asthma control in both clinical practice and clinical trials.

### Clinical assessment of asthma control

Current clinical approaches characterize asthma based on four defining domains; symptoms, airway inflammation, airway obstruction, and airway hyperresponsiveness. How are these domains assessed and how do these assessments reflect asthma control? Furthermore, how do these outcome measures compare to those available in murine models of allergic inflammatory airway disease?

### Domain 1: symptoms

Symptoms, including cough, wheeze, chest tightness, and dyspnea, can be measured using a variety of symptom diaries and questionnaires (Reddel et al., 2009). Items such as symptom-free days and reliever use are recorded and quality of life is assessed using questionnaires (Juniper et al., 1992; O'Byrne et al., 2005). The use of symptoms as indicators of underlying physiologic and immune status has been exploited through asthma action plans, which provide patients with the ability to assess and exert some level of control over symptom progression and reduce the need for medical resource intervention (Gupta et al., 2012; Lougheed et al., 2012). For obvious reasons assessment of symptoms is difficult in murine models, which tend to be strong on quantitative measures of immune, physiologic, and histologic status.

### Domain 2: inflammation

Inflammation can be measured directly through bronchoscopy and biopsy. However, these procedures are invasive, time consuming, costly, and require expertise. Thus, inflammatory biomarkers of asthma hold great allure, and have been keenly pursued. To date, biomarkers such as exhaled nitric oxide, induced sputum and serum eosinophils have proven useful in identifying asthmatics having a predominant eosinophilic inflammatory phenotype (Green et al., 2002; van Veen et al., 2008, 2009). Similarly, serum periostin appears to be a promising marker of Th2 inflammation in asthmatics (Arron et al., 2011). However, none of the current biomarkers of airways inflammation have proved clinically meaningful with respect to asthma control (Holgate, 2012). Thus, the search continues for a non-invasive biomarker of airway inflammation that can reliably and reproducibly characterize asthma control. Genetic and experimental murine models of asthma are strong on the potential to assess inflammatory status through BAL, blood, histology, and molecular mechanisms, although it has been suggested that such models may not translate as well as one would hope with respect to human health (Holgate and Polosa, 2008). Perhaps a key factor that was missing for the exploitation of murine models was a more precise classification of human asthma based on pathophysiological mechanisms. Improved characterization of human asthma into recently defined “endotypes” (Lötvall et al., 2011; Agache et al., 2012) coupled with murine models that better target human signs and symptoms have the potential to enhance further the translation/impact of murine models. Additionally, the power of the murine models is vested within the controlled genetic background of laboratory mice, the restricted and consistent animal care environment and an ability to induce single gene mutations to provide a genotype-phenotype assessment. This magnitude of control, although far from fully transparent with respect to genotype-phenotype associations, is far more precise than the continuum of environmental exposures experienced by human asthmatics with highly variable genetic backgrounds.

### Domain 3: lung function (airway obstruction and bronchodilator reversibility)

Lung function in asthma is assessed clinically using spirometric methods that rely on the forced vital capacity (FVC) and derived measures such as forced expired volume in 1 second (FEV1), forced expired flow from 25–75% vital capacity (FEF 25–75%), or peak expiratory flow (PEF) (Reddel et al., 2009; Lougheed et al., 2012). Although these measures are typically made in the laboratory, ambulatory PEF is also reliably self-measured in the patient. Despite the convenience of ambulatory PEF measurements, FEV1 and the FEV1/FVC ratio are currently the customary diagnostic spirometric measures used to characterize airflow limitation and bronchodilator (BD) responsiveness in asthmatics (Reddel et al., 2009; Lougheed et al., 2012). Although it is tempting to link spirometric FEV1 and FEV1/FVC measurements to airway or lung resistance (*R*_L_), this is a complex relationship and “test of forced expiration and the direct measurement of airway resistance should not be regarded as invariably interchangeable” (Pride, 1971). Indeed, thorough and still valid historical perspectives of the factors affecting FEV1 and resistance outline the dependence of FEV1 on lung elastic recoil as well as intrapulmonary airway resistance (Pride, 1971). Nevertheless, spirometry and the FEV1 and FVC measurements are components of the diagnostic mainstay from which clinicians gain insight into the degree of airway narrowing, an indicator of disease progression and severity. Airway narrowing, or the average airway lumen diameter, reflects the complex interactions among physiologic and pathophysiologic events including airway inflammation, remodeling, and smooth muscle shortening, the latter two being of greater determinative importance. Airway smooth muscle shortening, which reduces airway diameter (Cabezas et al., 1971), is determined by the bronchoconstrictor reactivity and/or sensitivity (“responsiveness”) of the muscle itself and the structural load against which the muscle must pull (McParland et al., 2003). Airway remodeling is a broad term that includes structural changes to the airway mucosa (i.e., goblet cell hyperplasia and mucus hypersecretion) and submucosa (i.e., reticular basement membrane thickening) along with thickening of the airway wall (i.e., ASM hypertrophy/hyperplasia) (McParland et al., 2003; Broide, 2008; Evans et al., 2009; GINA, 2011; Shifren et al., 2012). This structural remodeling results in encroachment of the airway lumen, the extent of which is reflected by the magnitude of “irreversible” or “fixed” airway obstruction (McParland et al., 2003; GINA, 2011; Shifren et al., 2012) A clue as to the relative contributions of ASM shortening and airway remodeling to airway narrowing can be derived from considering pre- and post-BD FEV1 values. Whereas a BD, usually a β_2_-adrenergic receptor (β_2_-AR) agonist, has no immediate effect on airway remodeling, it will relax airway smooth muscle thereby reducing airway narrowing. This BD-induced demonstration of rapidly reversible airflow obstruction is one of the hallmark features of asthma (GINA, 2011) and sheds light on the relative magnitude of reversible (ASM shortening) vs. fixed (remodeling) airway obstruction present in asthmatics (Bumbacea et al., 2004). However, interpretation of β-agonist-induced reversibility maneuvers may be confounded by β_2_-AR dysfunction which is prevalent in severe asthmatics (Newnham et al., 1994, 1995; Grove and Lipworth, 1995; Taylor, 2009) or by remodeling-induced changes to airway geometry that amplify the airway narrowing effect of bronchoconstrictor stimuli (Lambert et al., 1993; McParland et al., 2003).

Airway narrowing increases the resistance to airflow through the airways thereby “limiting” or “obstructing” airflow, leading to a fall in spirometric indices of lung function, as well as dynamic lung hyperinflation and loss of inspiratory capacity (Lougheed et al., 2006). This in turn increases the work of breathing and leads to an array of complex and incompletely understood mechanical and sensory factors that manifest as dyspnea, chest tightness or other symptoms (O'Donnell et al., 2009). Thus, the challenge of translating human asthma and its diagnostic assessment to murine models is significant (Holgate, 2012). In mice, voluntary maneuvers are impossible. Some investigators have used negative pressure expiration in mice to simulate a forced expired maneuver (Lai and Chou, 2000; Gebel et al., 2010; Vanoirbeek et al., 2010); however, the outcome measures derived from this technique have not been thoroughly validated. Despite this limitation, quantitative and reliable measures of *R*_L_ and elastance can be produced in mice as well as changes in lung volume (Vinegar et al., 1979; Martin et al., 1988).

### Domain 4: airway hyperresponsiveness

AHR is a broad term that reflects the degree of airway narrowing in response to a given concentration of bronchoconstrictor. Thus, by definition, AHR is a measure of the complex interactions between ASM shortening and airway remodeling. Although AHR is detected using the FVC technique, which provides standard measures of lung function, critical insight is reflected by the sensitivity and reactivity of the asthmatic's airway to the bronchoconstrictor. AHR, or bronchial hyperresponsiveness, is assessed by measuring changes in function (i.e., the drop in the FEV1) from baseline with increasing inhaled concentrations of “direct” smooth muscle contractile agonists (e.g., methacholine, MCh) or “indirect” bronchoconstrictor stimuli (e.g., mannitol, an osmotic stimulus, or cold air) (Brannan and Lougheed, 2012; Tepper et al., 2012). From the constrictor-induced changes in FEV1 one can measure or calculate (via log-linear interpolation) the provocative concentration of bronchoconstrictor that has caused, or will cause, a particular magnitude of airway narrowing, typically a 20% decline in FEV1 (PC20). AHR is a highly-standardized, objective and reproducible outcome measure (Reddel et al., 2009; Tepper et al., 2012).

### Alternative lung mechanics tests

By modifying commonly used measurement techniques, indices of lung mechanics can be further assessed. For example, hyperinflation, inferred from changes in lung volumes measured by spirometry, is indicative of gas trapping secondary to airway obstruction and may occur in asthmatics (for example see Lougheed et al., 2006). Additionally, combining whole body plethysmography with bronchial challenge testing in humans can provide, usually in a research setting, a measure of airway resistance (Tepper et al., 2012). An alternative technique to the resistance measurements obtained through body plethysmography is the use of the forced oscillation technique (FOT) (Dubois et al., 1956). In this technique a specialized pressure generating device is computer-programmed to produce multiple simultaneous variations in airflow frequency (input) and sensitive pressure transducers capture the respiratory system pressure response (output) to this perturbation. A comparison of the Fourier transformed input and output data with respect to frequency gives the respiratory system impedance, from which airway resistance and elastance are calculated (Bates et al., 2011). Although the FOT is not routinely used clinically, it offers one major advantage in that it can be easily used in patient populations that are unable to perform the forced expired maneuvers (like the elderly or very young) (Bates et al., 2011; Tepper et al., 2012). Interestingly, the FOT is the same as that used by some investigators to assess murine respiratory system mechanics (Schuessler and Bates, 1995; Irvin and Bates, 2003).

### Asthma control outcome measures

As mentioned previously, asthma control is characterized by a patient's recent clinical state and future risk of adverse events. The recent clinical state is assessed through characterization of symptoms, frequency of reliever use and lung mechanics data. Specifically, lung function and AHR measurements are made using pulmonary function tests such as pre- and post-BD FEV1, PEF, and AHR. Future risk, the second component of asthma control, is defined as the probability of adverse outcomes such as worsening of the clinical state, increased exacerbations and accelerated decline in lung function. Since exacerbations, are defined as “events characterized by a change from the patient's previous status,” they implicitly include the same measurements as those described above for clinical state. However, the frequency and intensity of exacerbations holds additional information for the clinician. Currently, human lung mechanics measurements (lung function and AHR) are the most important measures for assessing and predicting the course of asthma.

### Clinical relevance

A wide variety of outcome measures, including symptoms, inflammation, and lung mechanics, are available to clinicians to characterize the current state and progression of asthma. Busse et al., recently suggested that AHR should be the “therapeutic or interventional target” (Busse, 2010) and evidence suggests that the use of MCh-induced AHR as a guide to treatment improves asthma control (Sont et al., 1999). AHR is also predictive of the effectiveness of inhaled corticosteroid therapy to mitigate lung function changes in asthmatics (Brutsche et al., 2006). Taken together, AHR is an indirect, but consequential marker of asthma control. In fact, those diagnosed with asymptomatic AHR often go on to develop asthma (Brutsche et al., 2006). Alternatively, lung function, as measured by spirometry (FEV1) has a high predictive value of asthma control including asthma exacerbations and asthma-related death. Indeed, some clinicians opine that AHR, although informative, is only a secondary measure of importance relative to pre- and post-BD FEV1 (Tepper et al., 2012). The overarching conclusion drawn by the ATS task force is that the best measurements currently available to accurately assess asthma control in clinical practice or clinical trials are both lung function and AHR (Reddel et al., 2009).

Although somewhat counterintuitive, given that inflammation is a foundational component of the definition of asthma (Lougheed et al., 2012), inflammatory status is not listed in the guidelines as a primary indicator of asthma control (Reddel et al., 2009). Although airways inflammation likely contributes to the development of symptoms and airflow obstruction, it does not closely associate with AHR (Rosi et al., 1999; Haldar et al., 2008; Busse, 2010). Perhaps consequently, inflammatory biomarkers have not been validated as tools for assessing asthma control as they too are not consistently associated with lung mechanics outcome measures. In addition to lung mechanics and inflammation outcome measures, clinicians have available a battery of methods to assess symptoms. Symptom perception is a significant factor in lung diseases; however, evidence suggests that symptoms are not closely associated with AHR or, in some cases, lung function and are therefore, dissociated from asthma control (Lougheed, 2007). For example, Jenkins et al, showed that LABA therapy that results in improved symptoms does not correspondingly improve airway obstruction as measured by pre-BD FEV1 (Jenkins et al., 2005). The importance of lung function and AHR measurements to guide clinicians in their assessment of asthma control is well-supported and, by analogy, emphasizes the importance of making lung function/lung mechanics measures in mice that provide data with comparable physiologic meaning and insight into pathophysiologic mechanisms.

## Assessing outcome measures in murine models of asthma

### Symptoms and inflammation

The murine correlate to asthmatic signs and symptoms is lacking. Symptoms cannot be communicated by animals. Furthermore, signs of the disease are not reliably displayed by prey species such as mice, nor interpreted by investigators. Mice do not appear to be capable of cough (Pack et al., 1984). The challenges of assessing wheeze, chest tightness, dyspnea, or other stress-related symptoms in mice are high. However, stress has been shown to influence the outcome of murine models of asthma (Quarcoo et al., 2009; Vig et al., 2010) and tools are available to assess or account for animal behavior (Hurst and West, 2010). Although utilization of these tools to assess murine signs of disease is beyond the scope of the present review, they may provide an, as yet, untapped opportunity to address mechanisms of dyspnea (O'Donnell et al., 2009). However, evaluating signs of asthma in mice is not currently practical.

On the other hand, mouse models permit much greater procurement of immunological and histological data than human methods (Lin et al., 2012), although care must be taken to not over-interpret immunological outcomes that are not well-supported by lung mechanics outcomes. Mullane recently reviewed studies in which immunological mechanisms underlying the pathophysiology of asthma were elucidated using murine models (Mullane, 2011). The relevance of these pathways to asthma pathogenesis was evaluated through mouse phenotyping that included measurement of AHR and indices of inflammation. Mullane's analysis illustrated that approximately only one quarter of the immunologic targets identified in murine studies showed positive therapeutic effects when manipulated in asthmatics. Although this appears to be a low yield on the potential of immunological targets translated to human clinical trials, our reexamination of the murine models in which AHR was shown to be diminished in response to intervention at a particular immunologic target suggests a more promising outcome (Table 1). Reexamination of the data is consistent with the notion that much of the translational value of murine immunologic mechanistic studies may rely on the concomitant rigor of AHR measurements. For example, immunologic pathways that produced positive or negative results when targeted clinically were associated with murine studies where AHR measurements were made more (i.e., invasive measures using appropriate dose response protocols) or less (i.e., unmeasured or measured using PenH or invasively with no dose response) stringently, respectively. The low rate of translational success from murine models to human asthma is undoubtedly influenced by several factors other than the stringency of AHR measurements including species differences, timing of intervention in relation to disease progression, genetic and environmental heterogeneity of the asthmatic (vs. the murine) population and the suitability of acute animal modeling of a chronic disease. However, a prevailing hypothesis appears to be that mechanistic information derived from mouse models of asthma will be more relevant clinically (more translatable) if AHR measurements are more rigorous (see next section). Interestingly, this notion echoes the conclusion reached by an NIH expert group that reviewed pulmonary function techniques for asthma with respect to the benefit of standardized and rigorous measures of lung function in human asthma (Tepper et al., 2012). Similar standardization for lung mechanics measurements in murine models of asthma would be prudent.

**Table 1:** **The impact of AHR measurement methodology on translation of immunological targets is shown below**.

**Immune target**	**Murine AHR**	**Effect of clinical targeting**	**References**	**Method of AHR measurement**	**Bronchoconstrictor**
IL-1	↓	No	Wang et al., 2006	PenH, no validating measure	aerosolized MCh, 0–50 mg/mL
IL-10	↑	No	van Scott et al., 2000	Esophageal Ptp, flow pneumotach	i.v. MCh up to 1000 ug/kg
IL-10	↓	No	Stämpfli et al., 1999	End-inflation occlusion	i.v. MCh up to 1000 ug/kg
IL-12	↓	No	Gavett et al., 1995	APTI	i.v. Ach, single dose at 25 ug/kg
IL-12	↓	No	Kips et al., 1996; Brusselle et al., 1997	Needle Ptp, flow pneumotach	i.v. carbachol 20 to 400 ug/kg
IL-13	↓	No	Karras et al., 2007	PenH dose response, baseline included, single dose FOT	MCh aerosol, 0–100 mg/mL; i.v. MCh single point
IL-13	↓	No	Munitz et al., 2008	FOT; 2 cmH2O PEEP	aerosolized MCh, 25–100 mg/mL
IL-13	↓	No	Grünig et al., 1998	PC_200_RL, baseline not provided; APTI and flow plethysmography	i.v. Ach, dose not given
IL-13	↓	No	Wills-Karp et al., 1998	APTI	i.v. Ach, single dose at 50 ug/kg
VLA-4, original	↓	No	Koo et al., 2003	Eos in lung by BALF and histology, no AHR measure	N/A
CD11a	N/A	No	Rabb et al., 1994	Not measured	N/A
LTB4	N/A	No	Walsh and August, 2010	Not measured	N/A
LTB4	N/A	No	Waseda et al., 2011	Not measured	N/A
IFN	N/A	No	Iwamoto et al., 1993	Not measured	N/A
IL-4R	↓	Positive	Rankin et al., 1996	Flow-plethysmography, PC_100_RL	MCh aerosol, begin dose 0.001 mg/mL
IL-4R	↓	Positive	Corry et al., 1996	APTI and Flow-plethysmography	i.v. MCh, 5 pt dose response
IL-3/5 /GMCSF	↓	Positive	Allakhverdi et al., 2002	Flow-plethysmography, EC_200_LTD4	i.t. LTD4; 50 to 1000ng
IL-3/5 /GMCSF	↓	Positive	Allakhverdi et al., 2006	Flow-plethysmography, EC_200_LTD4	i.t. LTD4; 50 to 1000ng
IL-2	↓	Positive	Doganci et al., 2008	Flow-plethysmography; ED_200_RL	i.v. MCh 33 to 3300 ug/kg
Selectin	↓	Positive	De Sanctis et al., 1997	Flow-plethysmography; ED_200_RL	i.v. MCh 33 to 1000 ug/kg
IL-5	variable	Positive	Garlisi et al., 1999	AHR not measured	N/A
IL-5	variable	Positive	Mauser et al., 1993	ED_100_RL and ED_40_Cdyn	Substance P

*Note that the immunological mediators identified in studies where AHR was not measured produced no significant effect when targeted clinically. i.v., intravenous; i.t., intratracheal; PC100RL, provocative concentration causing a 100% change in lung resistance; LTD4, leukotriene D4; MCh, methacholine; N/A, not applicable*.

### Murine lung mechanics measurement techniques

Several excellent reviews of the experimental validation, practical applications, feasibility, and limitations of experimental techniques for measuring lung mechanics in mice are available in the literature (Irvin and Bates, 2003; Glaab et al., 2007). Here, we provide a brief summary of these techniques and a discussion of how they can be optimized and possibly standardized for use in murine models of lung disease.

In a remarkable foreshadowing of the explosion of murine models of asthma, Martin et al. (1988) alluded to the attractiveness of murine models in immunology and genetics coupled with an acute need for robust measures of pulmonary mechanics and airway responsiveness. They successfully scaled-down standard plethysmographic techniques for measuring *R*_L_ (and its reciprocal conductance) and dynamic pulmonary compliance (Cdyn) (and its reciprocal elastance) in larger animal models (Mead, 1961) to accurately assess these variables in the mouse during intravenous challenge with a range of bronchoconstrictor and BD agents. Indeed, this study represents the foundation for modern approaches to the measurement of murine lung function (Ewart et al., 1995; Volgyesi et al., 2000; Irvin and Bates, 2003; Glaab et al., 2007). The classic method of measuring pulmonary mechanics in mice requires the measurement of transpulmonary pressure, airflow and tidal volume from which the lung function parameters total *R*_L_ (the sum of frictional airflow and pulmonary tissue resistances) and Cdyn can be calculated [by fitting them to an equation of motion (Amdur and Mead, 1958; Mead, 1961; Irvin and Bates, 2003)].

$$P=V\times \text{1}/C+R_{\text{L}}\times \text{Flow}$$

(*P*, transpulmonary pressure; *V*, lung volume relative to functional residual capacity; *C*, compliance; *R*_L_, total lung resistance; Flow, air flow through the airways).

Thus, transpulmonary pressure, calculated as the difference between directly measured mouth and intrapleural pressures, has an elastic component (*V* × 1/*C*) and a flow-resistive component (*R*_L_ × Flow) and these can be evaluated separately. When gas flow ceases (at end-inspiration and end-expiration) the flow-resistive component of the equation drops out and compliance can be calculated based on *P* and *V*. To calculate *R*_L_, transpulmonary pressure and Flow are measured at points of equal volume during the respiratory cycle (where elastic pressures will be approximately equal) so that the transpulmonary pressure difference between these two points reflects total *R*_L_ (Amdur and Mead, 1958). As the equation suggests both airflow and transpulmonary pressure are required to calculate resistance. Given that mice have extremely small tidal volumes, airflow is difficult to measure unless appropriately sized pneumotachographs are constructed (Mortola and Noworaj, 1983); thus, the flow signal is often measured by electronic differentiation of the volume signal which is generated using the plethysmograph technique. Intrapleural pressure can be measured directly or indirectly by connecting a fluid-filled intrapleural catheter or an esophageal balloon, respectively, to a pressure transducer.

**Figure 1:**
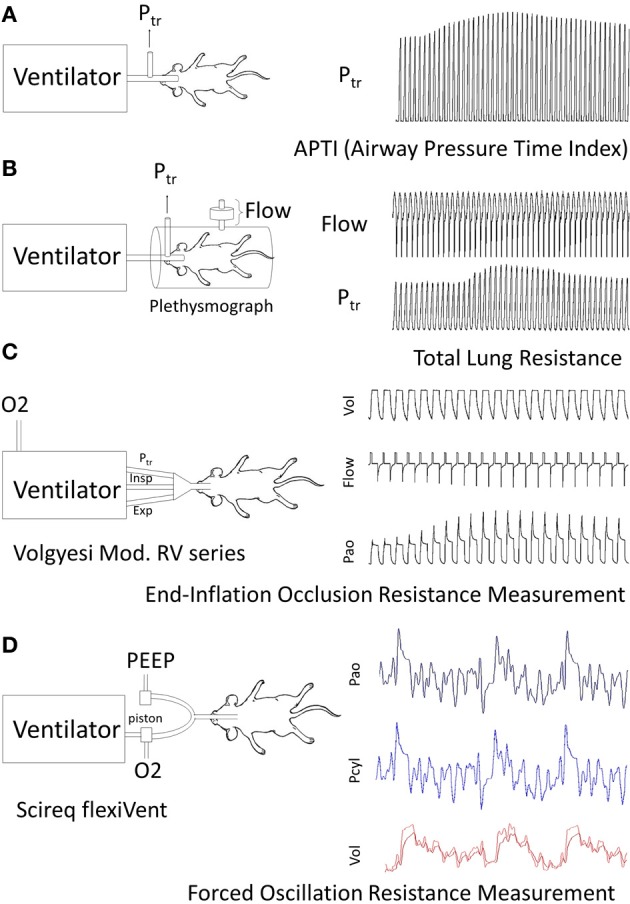
**Examples of routine methods of Respiratory mechanics Measurements for AHR: each panel illustrates animal instrumentation, airway measurement (s) and presence of mechanical or spontaneous ventilation. (A) Airway Pressure Time Index**: APTI measures the tracheal pressure response to MCh in a mouse to calculate an aggregate change in respiratory system impedance (Levitt and Mitzner, 1988). **(B) Flow Plethysmography**: Flow and tracheal pressure (P_tr_) signals derived from a flow-plethysmograph and tracheal cannula are used to calculate Rrs or RL depending on whether transrespiratory or transpulmonary pressure is measured (Amdur and Mead, 1958; Waldron and Fisher, 1988). **(C) End-Inflation Occlusion**: This technique relies on a purpose built ventilator to deliver and hold known inflation volumes and measure the resultant pressure peak and plateau pressures to calculate Rrs and Crs (Ewart et al., 1995; Volgyesi et al., 2000). Ventilators may be lab constructed or use a commercial product (Volgeysi ventilator). **(D) Forced Oscillation Technique**: The FOT method relies on a purpose built ventilator requiring sophisticated software for the control of volume perturbations and analysis of generated pressure, volume and flow signals. In brief, ventilator housed pistons generate a complex frequency perturbation in the volume signal administered to mice. The lung response is measured as pressure from which lung impedance and other variables are calculated. Current use applications in the literature appear to be restricted to a commercially built ventilator (Scireq®flexivent).

**Figure 2:**
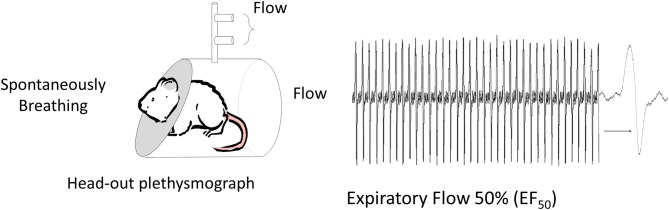
**Non-invasive method to measure respiratory mechanics.** Mid-Expiratory Flow: Flow is measured at 50% of the expired volume (EF_50_) from animals placed in a head-out plethysmograph. A pneumotach placed in the chamber, face mask (in humans) or tracheal cannula can be used to measure flow and volume (Vijayaraghavan et al., 1994; Glaab et al., 2001). This method provides an indirect assessment of resistance since the reduction in flow reflects any source of airway narrowing from pulmonary airways to changes in laryngeal adduction or even post inspiratory activation of the diaphragm.

Current methods that assess murine pulmonary function can be categorized broadly into invasive (Figures 1A–D) and non-invasive (Figure 2) techniques. Invasive techniques directly measure respiratory system (including chest-wall) or pulmonary (lung only) mechanics and typically rely on tracheal cannulation or orotracheal intubation to measure airflow and pressure across the respiratory system or across the lung (transpulmonary pressure). Based on physiological principles these methods provide reproducible, consistent, and meaningful data with respect to *R*_L_ and elastance, which are sufficiently sensitive to reveal subtle changes in lung mechanics induced by allergen-exposure models (Irvin and Bates, 2003; Glaab et al., 2007). Thus, invasive techniques are valuable tools for assessing AHR in murine models of asthma.

The primary drawback to the invasive methods, with the exception of the technically challenging orotracheal intubation method (Polikepahad et al., 2010; De Vleeschauwer et al., 2011), is the inability to make repeated measurements. A second disadvantage of measuring lung mechanics invasively is the need for anesthesia. Anesthesia will alter autonomic nervous system tone which may impact bronchoconstriction and/or mucin secretion (Dripps and Severinghaus, 1955; GINA, 2011). Whereas it is possible to make invasive lung mechanics measurements in conscious animals (Amdur and Mead, 1958), minor recovery surgery is required to place a fluid-filled catheter in the intrapleural space and significant restraint is required during measurements. Moreover, the challenge of making conscious lung mechanics measurements reflects what Amdur and Mead (Amdur and Mead, 1958) described as “the act of breathing” and the presence of movement and other artifacts that may yield a less sensitive measurement. For this very reason, non-invasive techniques, such as head-out body plethysmography (Figure 2) (Vijayaraghavan et al., 1994), that indirectly assess murine pulmonary function generate results that are less accurate and less reproducible than invasive measures (Glaab et al., 2007). Should non-invasive techniques evolve, it is important that they be validated against standard measures of lung and respiratory system mechanics prior to their widespread use (Mead, 1961; Irvin and Bates, 2003; Mitzner and Tankersley, 2003). The lack of such an approach leads to the adoption of techniques, which although attractive in application, may be neither valid nor appropriate experimentally (Irvin and Bates, 2003; Glaab et al., 2007). For example, the non-invasive technique for specific airway resistance originally developed by Pennock et al. (1979) relied on a well-recognized double chamber plethysmograph apparatus, in which signals from the thoracic (body) and head chamber allow one to obtain flow and pressure signals from which bronchoconstriction was estimated and this method was validated against traditional “gold standard” mechanics measurements. Subsequently, the Pennock method was modified and marketed as the PenH, or enhanced pause, method that claimed to provide an indirect measure of respiratory mechanics by placing a conscious unrestrained animal in a whole-body plethysmograph and simply measuring changes in chamber pressure (Hamelmann et al., 1997). The whole body plethysmograph approach, where the subject is placed within a sealed chamber, originated as a method to assess ventilation in conscious infants (Drorbaugh and Fenn, 1955) and has been validated for measurements of tidal volume and respiratory frequency based on variations in the pressure of the closed system according to the barometric method (Drorbaugh and Fenn, 1955). This method has been modified to accommodate other species including mice (Walker et al., 1997; Mortola and Frappell, 1998), for which several theoretical considerations have been elucidated for tidal volume measurement. PenH was not part of the above-mentioned developments of the barometric method to measure tidal volume and current evidence suggests that calculating PenH, is not a theoretically or biologically accurate measure of lung or respiratory system mechanics or of AHR (Drazen et al., 1999; Hantos and Brusasco, 2002; Bates and Irvin, 2003; Kips et al., 2003; Mitzner and Tankersley, 2003; Bates et al., 2004). Studies that correlate PenH with inflammatory indicators or other variables are, unfortunately, insufficient proof that PenH measures respiratory mechanics. Changes in PenH do however indicate that alterations in the pattern of breathing occur for conditions in which airway inflammation and possibly airway narrowing are present. This is not surprising given the rich array of airway afferents that are activated by inflammatory mediators (Fisher and O'Donnell, 2009; Fisher, 2011). Further, pattern of breathing changes can involve significant laryngeal resistance and abnormal upper airway laryngeal function can mimic asthma (Christopher et al., 1983; Morris and Christopher, 2012). An example of an “enhanced pause” that is not due to AHR or tracheobronchial airway narrowing is seen in newborn infants (Fisher et al., 1982; Mortola et al., 1982) where laryngeal adduction or closure, used to elevate lung volumes and clear pulmonary fetal liquid, leads to “enhanced pauses” that would provide erroneous conclusions about AHR based on PenH.

An appreciation for how the classic parameters (*R*_L_ and Cdyn) relate to lung structure and function in mice is not completely understood (Mitzner, 2007); however, in an idealized lung model airways resistance is directly linked to airway luminal diameter and airway closure can be quantified by measuring lung elastance (reciprocal of compliance) (Mitzner, 2007). Numerous invasive methods for determining *R*_L_ and Cdyn have been described above, each of which involves anesthetized and tracheally instrumented animals. Unless longitudinal measures of lung mechanics are the focus of a study, we recommend that these invasive techniques be the standard measure for assessment of murine lung mechanics. One such method that has gained recent attention in murine models of lung disease is the FOT which examines the frequency-domain of lung mechanics (Figure 1D). Although the FOT is not new (Dubois et al., 1956; Finucane et al., 1975), its application to murine models is relatively recent (Schuessler and Bates, 1995). The mathematical modeling underlying the FOT is complex, but has been well-described by others (Hildebrandt, 1970; Hantos et al., 1987; Schuessler and Bates, 1995; Bates et al., 2011) so will not be discussed here.

### Utility of lung mechanics techniques

Two divergent opinions exist with respect to the usefulness of classic vs. the FOT for measuring lung mechanics in mice (Bates, 2007; Mitzner, 2007). One school of thought is that the traditional measures, resistance and compliance, derived from the classic equation of motion (Mead, 1961) provide robust and reliable data to quantify AHR, baseline pulmonary mechanics and the impact of antigen-sensitization and challenge on them (Mitzner, 2007). Indeed, even airway pressure time index (APTI) (Figure 1A), a reflection of an aggregate change in respiratory system impedance, is sufficiently sensitive to reveal the effect of allergen-treatment on AHR (Walker et al., 2003). The other opinion is that additional mechanistic insight into airway structural changes can be generated by using complex mathematical modeling to examine how lung mechanics are influenced by frequency (Bates, 2007; Lundblad, 2012). Extension of this viewpoint claims that the FOT can provide (for both human and animal models) important physiological interpretations of disease progression and/or resolution associated with novel treatment strategies (Wagers et al., 2004, 2007; Lundblad et al., 2007; Schweitzer et al., 2010; Bates et al., 2011). Neither viewpoint is incorrect; as long as study designs embrace quantitative (and validated) measurements of lung mechanics as an outcome measure for murine models of inflammatory lung disease, then the methods employed largely reflect the goals of the investigator.

## Murine lung mechanics outcome measures

### Baseline resistance

**Figure 3:**
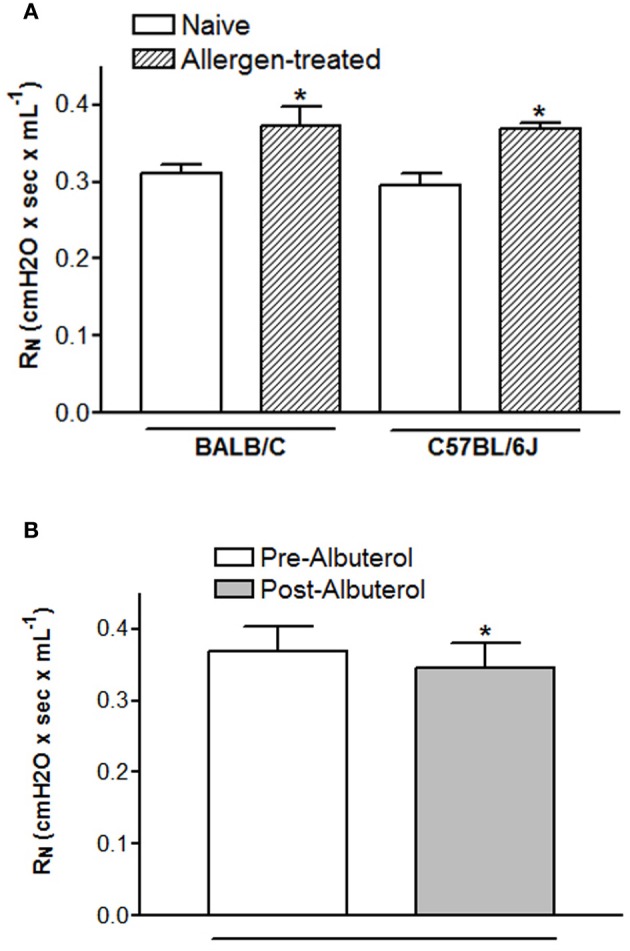
**Changes in baseline resistance are measureable in mice.** Baseline resistance was measured using the forced oscillation technique in anesthetized, paralyzed, tracheotomized mice 24 h after the last OVA challenge (Lin et al., 2012). Duplicate measures of baseline resistance were made in all mice. Animal experiments used to acquire these data were approved by the Duke IACUC. **(A)** and **(B)** BALB/C mice were sensitized by i.p. injection with 10 ug OVA adsorbed to 2.5 mg alum on days 0 and 14 and challenged by aerosol exposure with 1% OVA for 1 h on days 21, 22, and 23 (Walker et al., 2003). The effect of 60 ug/kg i.v. albuterol on baseline resistance was assessed 1 min after it was administered. **(B)** C57BL/6J mice were sensitized by i.p. injection with 10 ug OVA adsorbed to 2 mg alum on days 1 and 6 and challenged by intranasal insufflation 25 μL OVA (0.2% w/v in saline) containing 2.5 nM of short scrambled peptide. ^*^p < 0.05 from naïve **(A)** or pre-albuterol **(B)** as assessed by student's *t*-test.

The pre-BD FEV1 in humans reflects airway obstruction secondary to airway narrowing. The mouse correlate for determining airway narrowing is baseline airway resistance. In an idealized model, airway resistance mathematically reflects the luminal diameter of the airways (Mitzner, 2007). Murine baseline airway resistance is sufficiently sensitive to allergen treatment that changes can be detected using an acute ovalbumin (OVA) sensitization and challenge model. As shown in Figure 3A, acute OVA treatment increases baseline resistance relative to naïve controls for at least two strains of mouse (C57BL/6J and BALB/C). Comparison of the pre- and post-BD FEV1 in humans indicates the reversibility of airflow obstruction, which reflects the level of airway remodeling. Assessing the reversibility of airway tone can also be accomplished in mice by administration of a β_2_-AR agonist after initial baseline resistance has been measured. Figure 3B shows a statistically significant decline in baseline resistance immediately following i.v. delivery of 60 ug/kg albuterol in BALB/C mice. Despite receptor expression data suggesting that mice may mediate bronchorelaxation via β_1_-AR, it was recently shown that the bronchoprotective effect of intravenous (30 ug/kg) albuterol observed in wild type FVB/N mice is absent in β_2_-AR^−/−^ mutants (Lin et al., 2012). These data provide proof of concept that BD reversibility can be measured in mice. As in humans, measures of baseline resistance and BD reversibility may provide insight into the mechanisms underlying airway narrowing in mice, although correlation of these changes to airway remodeling or airway smooth muscle shortening has yet to be investigated. Additionally, airway remodeling studies necessitate the use of a chronic murine model of asthma, the characteristics and merits of which are reviewed in this issue by Kumar and Foster (Kumar and Foster, 2012).

### Airway hyperresponsiveness

AHR is a term that describes airway narrowing in response to a bronchoconstrictor challenge. The ASM shortening component of AHR consists of two main parts; increased sensitivity and increased reactivity. Increased reactivity refers to excessive airway narrowing in response to a contractile stimulus. Increased sensitivity describes airway narrowing that occurs in response to a smaller dose of bronchoconstrictor. To assess AHR in humans, FEV1 is measured after each exposure to increasing doses of constrictor. Similarly, increasing doses of MCh or other bronchoconstrictor are used in mice; however, airway or total *R*_L_ is measured instead of FEV1. Irrespective of differences in measurement techniques, lung mechanics measures are based on physiologic principles that apply across species suggesting that the pathophysiologic relevance of AHR measures in mice is translatable to humans, despite the inability to quantitatively compare the two. Thorough assessment of AHR requires examination of the response to several concentrations of bronchoconstrictor. To generate a typical dose response curve in mice, resistance measurements are taken at baseline and immediately after exposure to increasing doses of MCh. Airway resistance is plotted on the ordinate and the dose or concentration of constrictor on the abscissa. A leftward shift in the curve indicates increased sensitivity, whereas an increased slope of the curve represents increased reactivity. To calculate the slope, or reactivity, of the response to bronchoconstrictor one can apply non-linear regression analysis and fit the data to an exponential growth function (y = ae^kx^). Alternatively, the resistance data may be natural logarithm-transformed followed by linear regression analysis to provide a straight line, the slope of which is an indication of reactivity (Evans et al., 2003; Thanawala et al., 2013). It is thought that the increased reactivity component of AHR is more clinically relevant (Busse, 2010). Thus, an altered slope of the dose response curve in mice is the outcome that is likely more translatable to human asthma. Unfortunately, despite the acceptance of the notion that airway remodeling contributes at least in part to excessive airway narrowing, and thus, AHR, a definitive description of the location and nature of this remodeling is lacking (Busse, 2010). In addition, how the bronchoconstrictor is delivered (i.e., aerosol or vascular) can have an effect in human and animal models due to the health and integrity of the epithelium (Fisher et al., 1994; Turi et al., 2011) as well as the level of mucous hypersecretion (Wagers et al., 2007), distribution of blood flow (Holtzman et al., 1983) and the anatomical distribution of airway innervation (Cabezas et al., 1971). All of the invasive methods for measuring lung mechanics in mice discussed above have been validated as accurate indicators of AHR in mouse models of asthma.

## Translational utility of murine outcome measures

### Translation

Given how important human lung mechanics measurements (lung function and AHR) are to the assessment and prediction of the course of asthma, optimization of these measurements in mice would be beneficial. One example of successful translation of mouse models to human asthma is that regarding the controversy surrounding β-agonist use in asthma. Although β-agonists are the ultimate alleviators of acute bronchospasm, their chronic use can be associated with a number of adverse patient outcomes including loss of bronchoprotection, worsening of asthma control, and asthma-related death (reviewed in Walker et al., 2011). Despite the fact that some of these deleterious events were noticed more than 40 years ago, little advance has been made in the comprehension of the physiological effects of chronic β_2_-AR stimulation. Recently, Bond's group compiled a body of evidence indicating that β_2_-ARs mediate the development of allergic inflammatory airway disease in mice (Callaerts-Vegh et al., 2004; Nguyen et al., 2008, 2009). This group showed that β_2_-AR^−/−^ mice display a severe reduction in the asthma phenotype as do mice chronically treated with nadolol, a β_2_-AR inverse agonist. These studies embraced modern techniques of lung function assessment that mirrored those described for human asthma (Tepper et al., 2012). Using the FOT they generated multiple-point intravenous MCh dose-response curves and in separate groups of mice administered an i.v. bolus of salbutamol prior to MCh dosing to assess AHR and bronchoprotection, respectively (Callaerts-Vegh et al., 2004). Recently, two small clinical trials from the same group showed that chronic nadolol treatment was clinically safe and associated with reduced sensitivity to MCh as evidenced by a significant increase in PC20 values (Hanania et al., 2008, 2010). Currently, the effectiveness of nadolol as a treatment for asthma is being studied in a multi-center National Institute of Allergy and Infectious Diseases (NIAID)-funded Clinical Trial.

### Additional considerations for measuring and reporting murine AHR

AHR is one of the best clinical predictors for asthma control; thus, parallel measures in mouse models of asthma promise translational utility (Table 2). To examine both the sensitivity and reactivity components of AHR a multi-point dose-response curve characterized by incrementally increasing (i.e., doubling, quadrupling or half log increments) doses of bronchoconstrictor should be provided. The concentration of bronchoconstrictor that is physiologically relevant will depend on the nature of the bronchoconstrictor, but a dose that causes a 50% increase in baseline resistance is an acceptable midpoint for the curve. Reporting AHR data in absolute values is preferable for a variety of reasons. Firstly, baseline resistance reported in absolute values (either numerically or within a dose-response plot) provides a context for the bronchoconstrictor-induced changes as well as information about tonic airway narrowing. Secondly, reporting of absolute values affords investigators the opportunity to compare and contrast the effect of genetics on the lung mechanics responses to various disease models, as well as variations among similar protocols reported from different laboratories. While variability in resistance measures is expected, the lung mechanics of mice should, like humans, fall into a range of values since the fundamental structure and composition of the lung should not change within a strain/species. Finally readers can quickly infer percent change from the absolute data. However, if baseline values are different between groups or not reported, then it is more difficult or impossible, respectively, to make the reverse calculation. APTI is a measure that is only obtained during a bronchoconstrictor response and therefore, one cannot provide a “resting” or baseline value (Levitt and Mitzner, 1988). Stating that baseline resistance is not statistically significantly different between groups is less informative than reporting the absolute baseline values for each group.

**Table 2:** **Comparing lung mechanics measures in humans and mice**.

**Human asthmatics**			**Model of asthma**	
**Lung function implication**	**Human outcome measure**	**Human method**	**Murine outcome measure**	**Murine method**
Airflow obstruction	Pre BD-FEV1	Spirometry; FEV1/FVC compared to normals	Baseline resistance and/or lung volume.	Invasive lung mechanics; no bronchoreactive substance used; compared to non-treated controls
Airway remodeling (airflow obstruction reversibility)	Post BD-FEV1	Spirometry; FEV1/FVC post beta-agonist inhalation	Baseline resistance post-BD	Invasive lung mechanics; bronchodilator used
AHR	PC20 (direct constrictor) or PD20 (indirect constrictor) calculated	Spirometry; FEV1 pre- and post-increasing doses of bronchoconstrictor	Resistance; PC100 and/or slope of dose response curve	Invasive lung mechanics; doubling, quadrupling or half log increases in bronchoconstrictor concentration; regression analysis of dose response curve
BPE	Effect of bronchodilator on PC20; PC20 measured pre and post-intervention	Spirometry; FEV1 at baseline with increasing doses of bronchoconstrictor is measured pre- and post-BD	Resistance; PC100 and/or slope of dose response curve	Invasive lung mechanics; pre- and post-BD in same mouse or between groups comparison

*The table illustrates the translational utility of lung mechanics measures made in mice to those made in asthmatics. FEV1, forced expired volume in 1 second; PC20, provocative concentration of bronchoconstrictor that has caused a 20% decline in FEV1; BD, bronchodilator; FVC, forced vital capacity; MCh, methacholine; AHR, airway hyperresponsiveness; BPE, bronchoprotective effect*.

Many different bronchoconstrictor agents can be utilized in both human and murine AHR tests (Brannan and Lougheed, 2012). In mice, MCh is the most commonly used bronchoconstrictor and the route of delivery (intravenous or aerosol) may provide additional insight into the mechanisms underlying AHR (Wagers et al., 2007). For example, Wagers et al. have shown that when MCh is administered by inhalation, as opposed to an intravenous route, then airway mucous is more likely to contribute to airway closure which is observed as enhanced respiratory system resistance and elastance (Wagers et al., 2004). Similar to human studies, the use of nebulizers to aerosolize bronchoconstrictor agents to test AHR should be accompanied by details of the type of nebulizer and particle size generated, the duration of aerosol exposure and the timing between aerosol exposure and resistance measurements. Additionally, information such as the duration over which resistance measurements are made (in relation to timing of bronchoconstrictor administration) and the analysis method employed (average or peak) should be provided in order to interpret whether or not the relaxation phase or persistent elevation of resistance due to airway collapse, which immediately follows airway smooth muscle constriction, is contributing to the values reported.

Other methodological details such as those related to mechanical ventilation [positive end expiratory pressure (PEEP), breathing frequency and tidal volume] can, through their impact on lung volume and gas exchange, have a significant effect on lung compliance, airway resistance and the health of the animal. For example, others have shown in mice that baseline and bronchoconstrictor-induced airway resistances are inversely proportional to PEEP (Sly et al., 2003; Bates and Lauzon, 2007) and that the absence of PEEP in ventilated small mammals is likely to result in decreased lung compliance secondary to airway closure. The peripheral structure of the lung is much more complex than a single airway terminating at a single alveolus. Indeed, alveoli are interconnected not only with one another (Macklem, 1971), but with branching airways embedded throughout the parenchyma. The parenchymal tethering forces (radial traction) on the airways is beneficial with respect to airway patency; although there is a work of breathing trade off at high lung volumes due to reduced lung compliance. PEEP maintains airway pressure during normal passive exhalation, effectively increasing the volume of gas in the lungs relative to that at the end of a normal tidal breath (end-expiration). Because the mouse chest wall is highly flexible, conscious mice (and other smaller species) normally employ expiratory braking mechanisms or engage in frequency-induced dynamic hyperinflation to overcome naturally low end expiratory lung volumes and associated airway closure (Vinegar et al., 1979; Mortola, 1987). Thus, the loss of these mechanisms under anesthesia results in a decline in end-expiratory lung volume that can be restored to near conscious levels by PEEP. However, murine lung volumes are sensitive to PEEP and a 2–4 cmH_2_O increase in transpulmonary pressure has been reported to produce a doubling in absolute lung volume (Lai-Fook and Houtz, 2008). Another consideration of the effect of PEEP on end-tidal lung volume is how this relation will be influenced by lung structural elements. For example, a given level of PEEP will produce a lower volume in a fibrotic lung, than for a healthy lung. Thus, a constant level of PEEP does not necessarily equate with constant lung volume in lung disease models. In light of the above considerations, development of standardized methodologies that assess lung function in murine models of lung disease would enhance the ability to compare results between separate studies and improve interpretation of the data.

Given that one of the hallmark features of asthma is airflow obstruction, asthmatics experience dynamic hyperinflation (Lougheed et al., 2006) during episodes of acute bronchospasm. Unrelieved bronchoconstriction can promote incomplete exhalation, expiratory gas-trapping, and elevated end-expiratory volume. As mentioned previously, lung volume impacts the bronchoconstrictor response, thus interpretation of airway hyperresponsiveness is more informed when lung volume status is concomitantly considered. Although hyperinflation is not routinely examined during AHR measurements in mice, implementation of deep inspirations (to total lung capacity) following the cessation of bronchoconstrictor response measurements will relax airway smooth muscle and/or open collapsed airways to facilitate expiration and guard against hyperinflation (Tomioka et al., 2002). Similarly, it is recommended that deep inspirations be administered prior to delivery of any bronchoprovocative agent so that regions of atelectasis, which occur spontaneously and progressively over time (Mead and Collier, 1959), can be re-inflated. This maneuver ensures that the lung has a consistent mechanical starting point (i.e., control of volume history). Resistance and elastance can be measured between doses of bronchoconstrictor to ensure that these parameters return to control levels. These practices should be, but are rarely, reported in the methods section of original research articles.

The cardiovascular health of anesthetized mice is another factor that can affect the quality of lung mechanics data. For example, reduced cardiac output may alter the delivery of an intravenous constrictor or dilator to the airway smooth muscle. This consideration is illustrated by the enhanced bronchoconstrictor response to vascularly delivered muscarinic receptor agonists (acetylcholine and MCh) in M2-muscarinic receptor^−/−^ mutant mice, which lack the (sinoatrial nodal) M2-muscarinic receptor-mediated bradycardia observed in wild type controls (Fisher et al., 2004). AHR measures can also be impacted if treatment regimens or genetic deletions alter pulmonary perfusion since parenchymal airways in mice are perfused by pulmonary vessels/capillaries (Mitzner et al., 2000) rather than via systemic perfusion, as in humans. This unique murine anatomy makes the delivery of intravenous bronchoconstrictors and the circulatory clearance of any bronchoconstrictor, irrespective of its delivery method, susceptible to the hypoxic pulmonary vasoconstrictor response (Lundblad et al., 2007).

Although not typically measured in mice, bronchoprotective effectiveness is routinely assessed in asthmatics (Tepper et al., 2012) (Table 2). The bronchoprotective effect is defined as the effectiveness of a BD to protect the airways from constrictor-induced narrowing. This measure is distinct from BD effectiveness, or reversibility; however both are clinically relevant. The efficacy of a β_2_-AR-agonist to inhibit an induced bronchoconstriction is linked to asthma control in humans. Numerous studies demonstrate that loss of bronchoprotection occurs with chronic β-agonist use and that this is associated with worsening, or loss, of asthma control (Cheung et al., 1992; Bhagat et al., 1995; Drotar et al., 1998; Lipworth et al., 1998; Jokic et al., 2001). Consistent with the findings in human asthma, parallel results from several murine studies demonstrated a bronchoprotective effect of β_2_-AR-selective agonists (Callaerts-Vegh et al., 2004; Tamaoki et al., 2004; Riesenfeld et al., 2010; Lin et al., 2012). Taken together, murine models are appropriate for studies that examine the long-term effects of β_2_-AR ligands (short and long acting β-agonists, inverse agonists) on asthma pathogenesis or the effect of steroids on this receptor system.

## Summary and conclusion

The ATS recently assembled a task force charged with defining asthma control and determining the clinical outcome measures important for its assessment. The ensuing report clearly advocates that lung function and AHR be the principal outcome measures utilized in both clinical practice and clinical trials to guide treatment and evaluation of new therapies, respectively. Subsequently an expert panel concluded that spirometric outcome measures are of central importance in asthma and provided standards by which these measures should be assessed. We suggest that more robust assessment of lung mechanics in mice, both in methodology and scope, may provide mechanistic information that, coupled with modern cell and molecular biology techniques, will yield better clinical translation that many seek from fundamental science (Holgate, 2012). Included in this report are several specific recommendations with respect to how lung mechanics measures in mice might be approached and reported. Perhaps the most impactful suggestion we have made is that the utilization and reporting of murine lung mechanics measures and protocols become standardized.

### Conflict of interest statement

The authors declare that the research was conducted in the absence of any commercial or financial relationships that could be construed as a potential conflict of interest.

## Abbreviations

NAEPP: National Asthma Education Prevention Program
ATS: American Thoracic Society
AHR: Airway hyperresponsiveness
FVC: Forced vital capacity
FEV1: Forced expired volume in 1 second
FEF 25–75%: Forced expired flow from 25–75% vital capacity
PEF: Peak expiratory flow
β_1_-AR: β_1_-adrenergic receptor
β_2_-AR: β_2_-adrenergic receptor
BD: Bronchodilator
MCh: Methacholine
PC20: Provocative concentration of bronchoconstrictor causing a 20% decline in FEV1
PenH: Enhanced pause
*R*_L_: Lung resistance
FOT: Forced oscillation technique
Cdyn: Dynamic pulmonary compliance
*V*: Lung Volume
*P*: Transpulmonary pressure
OVA: Ovalbumin
NIAID: National Institute of Allergy and Infectious Diseases
APTI: airway pressure time index
PEEP: Positive end expiratory pressure.
